# Transcranial doppler sonography is not a valid diagnostic tool for detection of basilar artery stenosis or in-stent restenosis: a retrospective diagnostic study

**DOI:** 10.1186/s12883-017-0872-8

**Published:** 2017-05-11

**Authors:** Woori Koh, Kai Kallenberg, André Karch, Tobias Frank, Michael Knauth, Mathias Bähr, Jan Liman

**Affiliations:** 1 Department of Neurology, University of Goettingen, Robert-Koch-Str. 40, 37075 Goettingen, Germany; 2 Department of Neuroradiology, University of Goettingen, Robert-Koch-Str. 40, 37075 Goettingen, Germany; 3 Epidemiological and Statistical Methods, Department of Epidemiology, Helmholtz Centre for Infection Research, Inhoffenstr. 7, 38124 Braunschweig, Germany

**Keywords:** Doppler sonography, Duplex sonography, Basilar artery stenosis, In stent restenosis

## Abstract

**Background:**

There are contradictory reports concerning the validity of transcranial sonography (TCD and TCCS) for examinations of the basilar artery. Here we investigated sensitivity and specificity of transcranial sonography for the detection of basilar artery stenosis and in-stent-restenosis compared to cerebral angiography.

**Methods:**

We analyzed data of 104 examinations of the basilar artery. The association between sonographic peak systolic velocity (PSV) and degree of stenosis obtained by cerebral angiography was evaluated applying Spearman’s correlation coefficient. Receiver Operating Characteristics (ROC) curves and areas under the curve (AUC) were calculated for the detection of a ≥50% stenosis defined by angiography. Optimal cut-off was derived using the Youden-index.

**Results:**

A weak but statistically significant correlation between PSV and the degree of stenosis was found (*n*=104, rho=0.35, *p*<0.001). ROC analysis for a detection of ≥50% stenosis showed an AUC of 0.70, a sensitivity of 74.0% and a specificity of 65.0% at the optimal cut off of 124 cm/s. Results were consistent when analyzing examinations done in stented and unstented arteries separately (TCD VS DSA/CTA in unstented artery: AUC=0.66, sensitivity 61.0%, specificity 65.0%, TCD/TCCS VS DSA in stented artery: AUC=0.63, sensitivity 71.0%, specificity 82.0%). Comparing TCCS measurements exclusively to angiography, ROC analysis showed an AUC of 1.00 for the detection of an in-stent-restenosis ≥50% with a sensitivity and specificity of 100% when a PSV of 132 cm/s was used as a cut off value.

**Conclusion:**

Validity of TCD in the assessment of basilar artery stenosis or in-stent restenosis is poor. First results for TCCS are promising, but due to the small samplesize further studies with larger samples sizes are warranted.

## Background

Ischemic stroke is one of the leading causes of death worldwide [1]. Subdivided by vascular territories, strokes within the vertebrobasilar territory (about 30% of all strokes), and especially in the basilar artery territory (about 10% of all strokes) are less common than strokes in the anterior circulation [2]. Acute cerebrovascular events in the basilar artery territory, however, are, if not diagnosed and treated immediately, associated with the highest rate of morbidity and mortality of all stroke types. As basilar artery stenosis is one of the key risk factors for a stroke in the basilar artery territory, an easily applicable and valid diagnostic tool for the detection and follow up of basilar artery stenosis is needed. Today, digital subtraction angiography (DSA) is the gold standard for the diagnosis of basilar artery stenosis. In recent years computed tomographic angiography (CTA) has been proposed as a diagnostic tool for the detection of basilar artery stenosis, which has been shown to have non-inferior accuracy to DSA. [3]. However, both methods are invasive and thus share common limitations and complications (e.g. radiation, contrast agent application). While these limitations may be acceptable in acute life-threatening situations it would be beneficial to apply accurate and less invasive methods for follow up evaluations or bedside at intensive care units. It has been shown previously, that sonographic examinations such as Duplex and Doppler ultrasound have a high sensitivity and specificity in the diagnosis of carotid artery stenosis [4, 5] – making non-invasive and low risk diagnostic tools available for the anterior circulation. In our experience Transcranial Doppler Sonography (TCD) is also widely used as a screening tool for basilar artery stenosis and follow up examination after basilar artery stenting due to its simplicity of use and the good penetration into the soft tissue of the neck. But, studies evaluating its diagnostic accuracy showed contradictory results and either lacked a sufficient number of patients, or applied this diagnostic tool under optimized control conditions [3, 6–14]. The aim of the current study was to evaluate systematically the sensitivity and specificity of transcranial sonography as a diagnostic tool for the detection of basilar artery stenosis in comparison to the gold standards DSA and CTA in stented and unstented basilar arteries as well as to DSA after stent implantation in a typical clinical setting [15].

## Methods

### Patient population

**Fig. 1:**
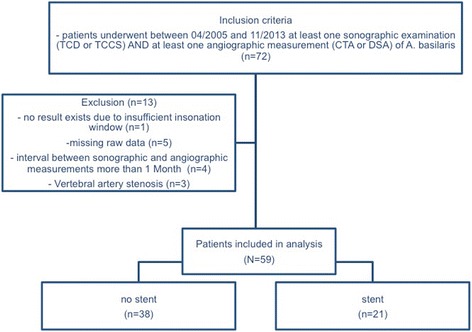
Flow chart showing the inclusion and enrolment criteria

**Fig. 2:**
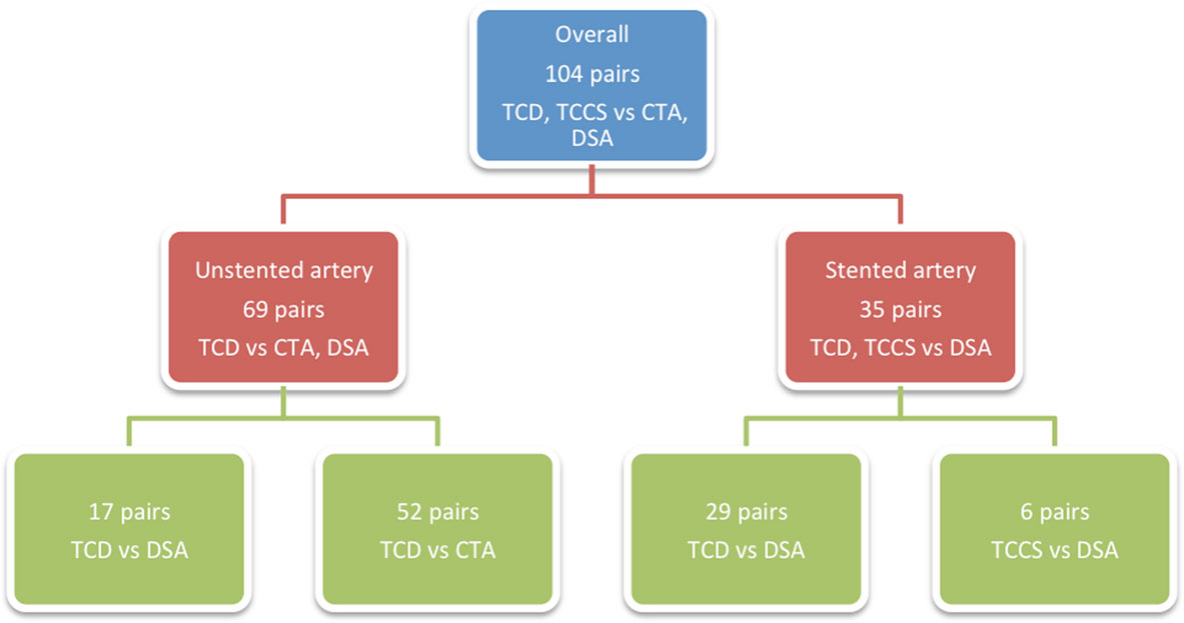
A diagram of number of pairs for each comparison

This is a retrospective diagnostic study using data from all patients (59) who received at least one sonographic examination (TCD or transcranial color-coded sonography (TCCS)) and at least one angiographic measurement (DSA or CTA), during routine work-up for suspected intracranial stenosis and/or a follow up examination after acute cerebral ischemic events in our tertiary care hospital between April 2005 and November 2013 (38 men and 21 women; mean age 68.6 (SD: 13.1 years), range 21 to 89 years) (for overview please view Fig. 1). Patients with combined vertebral and basilar artery stenosis were excluded from the study. The ethical committee of Goettingen University approved this project (No.: 8/11/11 An). Patients were excluded from the study if the interval between the examination modalities exceeded one month. The average interval between ultrasound and angiography examinations was 3.4 days (SD: 3.7 days, range: 0 to 20 days). Twenty-seven patients received more than one sonographic or angiographic examination during the routine work up and/or follow up examinations. We counted the number of pairs of sonography/angiography resulting in a total of 104 pairs. A subgroup of 21 patients underwent basilar artery stenting. From the follow up examinations of these patients a total of 35 pairs of sonographic and angiographic results were available (Fig. 2). Furthermore, we compared the results of CTA and DSA of the patient group without stenting using the intraclass correlation coefficient, in order to evaluate how strongly the measurements of these two methods resemble each other (13 pairs).

### TCD/TCCS

TCD was performed with a Multi Dop pro (DWL Elektronische Systeme GmbH, Singen, Germany) with a frequency of 2 MHz. Insonation depth was 80 mm to 120 mm. The TCCS examination was performed on an Acuson Sequoia (Siemens, Erlangen, Germany) according to standard procedures (2.5 MHz, 3 V2 transcranial imaging (TCI) probe with pulsed wave mode, mechanical index (MI) < 1.9, D-color starting with a Pule Repetition Frequency (PRF) of 0.30). During these examinations the peak systolic velocities (PSV), and end diastolic velocities (EDV) were obtained in consecutive insonation steps starting from 80 mm until the signal was lost or the insonation depth of 120 mm, as it is suggested by the german society for ultrasound in medicine (DEGUM) for transforaminal TCD of the basilar artery. Only the PSV measurements were used for further analysis. All sonographic measurements of the basilar artery were performed through transforaminal insonation in either sitting or lying position. All examinations were performed by neurologists (partly DEGUM certified) and experienced medical technicians, all examinations were supervised by a DEGUM certified neurologist. For the study all original ultrasound pictures were reevaluated by a DEGUM certified neurologist.

### CT-angiography (CTA), DSA

CT-angiography was performed on a 128-section multi-detector CT scanner (Definition AS+; Siemens, Erlangen/Germany; 120 kV, 120 reference mAs, rotation time 0.3 seconds, pitch 0.6, collimation 2×64×0.6 mm) after the injection of 65 mL of contrast in an antecubital vein applying a biphasic protocol (45 mL at 6 mL/s, 15 mL at 3 mL/s of a highly concentrated contrast agent with 400 mg iodine/mL), followed by a 30 mL saline chaser at 3 mL/s. CTA was acquired with automatic bolus triggering in the aortic arch (100 HU). Data were reconstructed with a section width of 0.75 mm. In addition to axial thin-section CTA source images, maximum intensity projections in axial and coronal planes were reconstructed (10-mm section thickness, 3-mm increment).

Biplanar DSA (Axiom Artis dBA; Siemens, Erlangen/Germany) examinations were carried out by fellowship-trained interventional neuroradiologists according to a standard operating procedure with guidelines heeding the patient’s clinical condition and anatomy. The studies were routinely reviewed on the angiography console in at least 2 orthogonal planes (typically posterior-anterior and lateral projections). Additional views were obtained whenever necessary.

Two readers (>12 years of experience in acute stroke imaging) blinded to the Doppler/Duplex results assessed the basilar artery stenosis according to the North American Symptomatic Carotid Endarterectomy Trial (NASCET, normal lumen diameter-residual lumen/normal lumen diameter) on a dedicated workstation by consensus reading. In cases of uncertainty regarding the stenosis grading on CTA images additional post-processing of the source data was performed on the manufacturer’s workstation (Leonardo, Siemens, Erlangen/Germany) applying the 3D tool.

### Statistical analysis

For statistical analysis SPSS 22 (SPSS Inc., Chicago, IL, USA) was used. The different diagnostic tools were compared in a two-step process; first, spearman correlation coefficients were used for investigating correlation between methods. In a second step, Receiver Operating Characteristics (ROC) curves and areas under the curve (AUC) were calculated (95% confidence interval is displayed). The optimal cut-off was derived using the Youden-index and sensitivities and specificities were determined for the cut-off value.

## Results

Agreement between the two angiographic gold standards CTA and DSA was compared using the intraclass correlation coefficient. There were a total of 13 pairs of measurements for comparison of unstented basilar artery stenosis available. The results showed a very good agreement concerning the degree of basilar artery stenosis between these two methods (ICC=0.87, 95% CI: 0.62–0.96). Based on these results and earlier studies [16, 17] the results obtained with CTA and DSA were regarded as equivalent and represented the gold standard for all further analyses.

All results obtained by sonographic methods (TCD, TCCS) were compared to those obtained by angiographic measurements (CTA, DSA) (n=104). Although there was some evidence for an association of angiographic and sonographic methods in the correlation analysis (rho=0.35, p<0.001), ROC analysis revealed a small area under the curve (AUC) of 0.70 (CI: 0.60–0.80) when basilar artery stenosis was defined as ≥50% in the CTA/DSA. A PSV of 124 cm/s was defined as the best cut-off by Youden-index resulting in a sensitivity of 74.0% and a specificity of 65.0%. When a stenosis of ≥70% was used as the reference an even smaller AUC was obtained by ROC analysis (AUC = 0.64 (CI: 0.49–0.79)).

The measurements were analyzed in more detail as reported below (Table 1).

**Table 1:** Measures of diagnostic validity and optimal PSV cut off values for the detection of ≥50% basilar artery stenosis using TCD and TCCS

Comparison	N	PSV cut off cm/s	Sensitivity %	Specificity %	AUC (CI 95%)
TCD, TCCS vs CTA, DSA	104	124	74.0	65.0	0.70 (0.60–0.80)
Unstented artery					
TCD vs CTA, DSA	69	146	61.0	65.0	0.66 (0.53–0.79)
TCD vs DSA	17	145	54.0	100.0	0.73 (0.49–0.97)
TCD vs CTA	52	165	30.3	95.0	0.65 (0.50–0.80)
Stented artery					
TCD, TCCS vs DSA	35	123	71.0	82.0	0.63 (0.35–0.91)
TCD vs DSA	29	123	67.0	78.0	0.56 (0.25–0.87)
TCCS vs DSA	6	132	100	100	1.00^a^

^a^CI not derivable

### TCD in unstented arteries

Sixty-nine measurements of PSV in the basilar artery obtained by TCD were compared to results obtained by angiographic measurements (CTA, DSA) in terms of degree of stenosis. Despite some evidence for a correlation (rho=0.25, p=0.042) ROC analysis for the detection of a basilar artery stenosis of ≥50% by TCD resulted in an AUC of 0.66 (CI: 0.53–0.79). The highest sensitivity (61.0%) and specificity (65.0%) was achieved with a PSV of 146 cm/s. The AUC of a ROC curve for detection of a stenosis of ≥70% showed similar results (AUC: 0.63 (CI: 0.46–0.80)).

### TCD and TCCS after stenting

In the subgroup of patients who underwent basilar artery stenting measurements obtained with DSA were used as the gold standard. A total of 35 pairs of DSA and sonographic measurements (TCD/TCCS) were available. There was only weak evidence for a correlation between DSA and TCD/TCCS measurements (rho=0.20, *p*=0.25). ROC analysis for detecting an in-stent-restenosis ≥50% by means of sonographic measurements showed an AUC of 0.63 (CI: 0.35–0.91). Best results were obtained using a PSV cut off of 123 cm/s with a sensitivity of 71.0% and a specificity of 82.0%. The AUC of the ROC curve for ≥70% in-stent-restenosis was 0.42 (CI: 0.00–0.92).

For a more detailed analysis results of TCD and TCCS were compared separately to DSA. Twenty-nine measurements of TCD were compared to measurements of DSA. No evidence for a correlation between the two methods could be observed (rho=0.13, *p*=0.50). The AUC of the ROC curve for the detection of an in-stent-restenosis ≥50% was small (AUC: 0.56 (CI: 0.25–0.87)). The PSV cut off with the highest Youden-index was 123 cm/s with a sensitivity of 67.0% and a specificity of 78.0%.

A total of 6 TCCS measurements were available for comparison with DSA. Again, there was a weak, but not significant correlation between these two methods (rho=0.37, p=0.47). Nevertheless for the detection of an in-stent-restenosis ≥50% ROC analysis showed an AUC of 1.00 with a sensitivity and specificity of 100% when a PSV of 132 cm/s was used as a cut off.

## Discussion

In this study the validity of transcranial sonography as a tool for detection of basilar artery stenosis and assessment of degree of stenosis in unstented as well as stented basilar arteries was evaluated.

### Usefulness of TCD in assessment of unstented basilar arteries

To date, DSA is the gold standard in evaluating basilar artery stenosis due to its excellent accuracy. However, because of the invasive character of the procedure with its known complications and limited availability, there have been attempts to find alternative diagnostic tools. As possible alternative modalities in the last decades CTA and sonographic methods were assessed. In previous studies it has been reported that CTA has a comparable accuracy in assessment of posterior circulation when compared to DSA and nowadays is widely used for this purpose [3, 11]. However, due to the radiation and contrast agent with nephrotoxic properties, especially for routine follow up examination this technique shows major drawbacks. Thus sonography based follow up examinations would be of great interest. But, evaluation of the validity of Doppler sonography for the assessment of basilar artery patency compared to angiographic methods showed conflicting results. Most of the done studies only had small numbers of patients, or applied this method on healthy volunteers with probably excellent anatomical prerequisites whereas our measurements were performed under routine conditions with patients of normal shape and with common limitations as for example short neck, agitation of the patient due to the cerebral infarction, or obesity [3, 6–14]. For example, in contrast to our findings, Tettenborn and colleagues showed a high sensitivity and specificity of TCD in the assessment of the vertebrobasilar artery in 48 patients compared to angiographic measurements, however there was no specific subgroup analysis for the basilar artery and the study only distinguished in a binary way between open vs. closed [8]. In our study we evaluated the accuracy of TCD for detection of a certain degree of stenosis. Therefore the two studies have a different approach and can only be compared to a limited extent. We did not find a clear correlation between the PSV obtained by TCD and the angiographic measurements (DSA and CTA) of the degree of stenosis. Furthermore, ROC analyses showed very low sensitivity for TCD in detecting stenosis of >50% and >70% indicating that TCD is not a valid tool for the detection of the degree basilar artery stenosis. This result is of interest, as the DEGUM in the latest version of 2011 besides TCCS is still recommending TCD for the detection of basilar artery stenosis.

The poor sensitivity of TCD for detection of basilar artery stenosis is in contrast, to validations of TCD in the evaluation of carotid artery stenosis with excellent results [4, 5]. However, it has to be kept in mind that the carotid artery is easily accessible and is located superficially whereas the basilar artery runs deeper being covered by connective tissues of the neck. In addition, anatomic variations are common in the posterior circulation [18] and the progression of degenerative vessel wall changes lead to elongation of the arteries causing unpredictable tortuosity and pathologic geometric variations [19]. These aspects result in more difficulties when assessing the basilar artery without any optical control, as the insonation angle is an important aspect when evaluating blood flow velocities. This explains differences in results between TCD to previous studies applying TCCS [14, 20, 21]. Another reason for the low sensitivity of TCD in detecting basilar artery stenosis in comparison to CTA/DSA could be changes in the degree of stenosis due to plaque rupturation in the time interval between the to examinations . In fact this is unlikely to be the cause, as not always the TCD was employed before the CTA/DSA and as some of the patients already were in a chronic phase of their artery disease and examinations were partly done for follow up reasons.

### Usefulness of TCD and TCCS in stented basilar artery

CT- and MR based follow up examinations of stented arteries, especially with small stent lumina have critical limitations in detecting the true lumen within the Stent. This is due to artefarcts caused by the stent material itself. TCCS though has proven to be of great value for the detection of in stent stenosis in the carotid artery [22–24]. Due to this convenience, transcranial sonography has also been applied to detect in-stent restenosis for follow up examinations of patients who previously underwent basilar artery stenting. To our knowledge, there are no studies analyzing the accuracy of TCD in detection of basilar artery in-stent restenosis and there is only one report of two cases, which evaluated the accuracy of TCCS in comparison to angiographic methods with promising results [25]. In line with our findings from unstented arteries, TCD did not qualify for the evaluation of in-stent restenosis. However, agreeing with the case report, the ROC analysis for TCCS showed a sensitivity and specificity of 100% for detection of ≥50% in-stent restenosis. This suggests that TCCS in contrast to TCD could be a valid method for the detection of in-stent restenosis. This can be explained by the fact that TCCS allows an optical control of angle and depth of insonation and anatomical structures, which TCD does not. Nevertheless the sample size for this particular analysis was very small in our study. Further analyses with larger case numbers will be necessary to confirm these findings. Other limitations of our study is the retrospective design, which does not allow to correct for possible confounders. For this reason also the inter examiner concordance could not be estimated.

The innovative aspect of our study is the examination and comparison of TCD, TCCS, CTA and DSA under routine circumstances. This provides valuable information about the effectiveness of the methods and clearly shows that TCD is very vulnerable to bad preconditions, which often occur within the clinical workflow. This information is of relevance, as to our knowledge TCD is still widely used as a routine application especially for insonation of the basilar artery due to its simplicity and excellent tissue penetrance. TCCS however showed promising results in basilar artery stenosis evaluation even under routine conditions.

## Conclusion

In summary, the validity of TCD in the assessment of the degree of basilar artery stenosis or in-stent restenosis is poor. Therefore, TCD is not suitable as a diagnostic tool in these settings. TCCS in contrast showed excellent sensitivity and specificity in detecting in-Stent stenosis of the basilar artery. Even though the number of examinations in our study was small and needs to be confirmed in larger samples, TCCS should be preferred for detection of stenosis in the basilar artery.

## Abbreviations

AUC: Areas under the curve
DSA: Digital subtraction angiography
PSV: Peak systolic velocity
ROC: Receiver operating characteristics curves
TCCS: Trancranial colour coded sonography
TCD: Transcranial dopplersonogrphy

## References

[1] Collaborators GMaCoD (2015). Global, regional, and national age-sex specific all-cause and cause-specific mortality for 240 causes of death, 1990-2013: a systematic analysis for the Global Burden of Disease Study 2013. Lancet.

[2] Chung JW, Park SH, Kim N, Kim WJ, Park JH, Ko Y, Yang MH, Jang MS, Han MK, Jung C, Kim JH, Oh CW, Bae HJ. Trial of ORG 10172 in Acute Stroke Treatment (TOAST) classification and vascular territory of ischemic stroke lesions diagnosed by diffusion-weighted imaging. J Am Heart Assoc. 2014;3 10.1161/JAHA.114.001119 PMC4310410 25112556

[3] Graf J, Skutta B, Kuhn FP, Ferbert A (2000). Computed tomographic angiography findings in 103 patients following vascular events in the posterior circulation: potential and clinical relevance. J Neurol.

[4] Wardlaw JM, Chappell FM, Best JJ, Wartolowska K, Berry E (2006). Non-invasive imaging compared with intra-arterial angiography in the diagnosis of symptomatic carotid stenosis: a meta-analysis. Lancet.

[5] Nederkoorn PJ, van der Graaf Y, Hunink MG (2003). Duplex ultrasound and magnetic resonance angiography compared with digital subtraction angiography in carotid artery stenosis: a systematic review. Stroke.

[6] Winter R, Biedert S, Reuther R (1984). Doppler sonogram in basilar artery thromboses. Eur Arch Psychiatry Neurol Sci.

[7] Ringelstein EB, Zeumer H, Poeck K (1985). Non-invasive diagnosis of intracranial lesions in the vertebrobasilar system. A comparison of Doppler sonographic and angiographic findings. Stroke.

[8] Tettenborn B, Estol C, De Witt LD, Kraemer G, Pessin MS, Caplan LR (1990). Accuracy of transcranial Doppler in the vertebrobasilar circulation. J Neurol.

[9] Mull M, Aulich A, Hennerici M (1990). Transcranial Doppler ultrasonography versus arteriography for assessment of the vertebrobasilar circulation. J Clin Ultrasound.

[10] Rorick MB, Nichols FT, Adams RJ (1994). Transcranial Doppler correlation with angiography in detection of intracranial stenosis. Stroke.

[11] Brandt T, Knauth M, Wildermuth S, Winter R, von Kummer R, Sartor K, Hacke W (1999). CT angiography and Doppler sonography for emergency assessment in acute basilar artery ischemia. Stroke.

[12] Cantu C, Yasaka M, Tsuchiya T, Yamaguchi T (1992). Evaluation of the Basilar Artery Flow Velocity by Transcranial Doppler Ultrasonography. Cerebrovasc Dis.

[13] Zhao L, Barlinn K, Sharma VK, Tsivgoulis G, Cava LF, Vasdekis SN, Teoh HL, Triantafyllou N, Chan BP, Sharma A, Voumvourakis K, Stamboulis E, Saqqur M, Harrigan MR, Albright KC, Alexandrov AV (2011). Velocity criteria for intracranial stenosis revisited: an international multicenter study of transcranial Doppler and digital subtraction angiography. Stroke.

[14] Kermer P, Wellmer A, Crome O, Mohr A, Knauth M, Bahr M (2006). Transcranial color-coded duplex sonography in suspected acute basilar artery occlusion. Ultrasound Med Biol.

[15] Struffert T, Ott S, Adamek E, Schwarz M, Engelhorn T, Kloska S, Deuerling-Zheng Y, Doerfler A (2011). Flat-detector computed tomography in the assessment of intracranial stents: comparison with multi detector CT and conventional angiography in a new animal model. Eur Radiol.

[16] Bash S, Villablanca JP, Jahan R, Duckwiler G, Tillis M, Kidwell C, Saver J, Sayre J (2005). Intracranial vascular stenosis and occlusive disease: evaluation with CT angiography, MR angiography, and digital subtraction angiography. AJNR Am J Neuroradiol.

[17] Nguyen-Huynh MN, Wintermark M, English J, Lam J, Vittinghoff E, Smith WS, Johnston SC (2008). How accurate is CT angiography in evaluating intracranial atherosclerotic disease?. Stroke.

[18] Ringelstein EB, Kahlscheuer B, Niggemeyer E, Otis SM (1990). Transcranial Doppler sonography: anatomical landmarks and normal velocity values. Ultrasound Med Biol.

[19] Han HC (2012). Twisted blood vessels: symptoms, etiology and biomechanical mechanisms. J Vasc Res.

[20] Martin PJ, Evans DH, Naylor AR (1995). Measurement of blood flow velocity in the basal cerebral circulation: advantages of transcranial color-coded sonography over conventional transcranial Doppler. J Clin Ultrasound.

[21] Schoning M, Buchholz R, Walter J (1993). Comparative study of transcranial color duplex sonography and transcranial Doppler sonography in adults. J Neurosurg.

[22] Robbin ML, Lockhart ME, Weber TM, Vitek JJ, Smith JK, Yadav J, Mathur A, Iyer SS, Roubin GS (1997). Carotid artery stents: early and intermediate follow-up with Doppler US. Radiology.

[23] Ringer AJ, German JW, Guterman LR, Hopkins LN (2002). Follow-up of stented carotid arteries by Doppler ultrasound. Neurosurgery.

[24] AbuRahma AF, Abu-Halimah S, Bensenhaver J, Dean LS, Keiffer T, Emmett M, Flaherty S (2008). Optimal carotid duplex velocity criteria for defining the severity of carotid in-stent restenosis. J Vasc Surg.

[25] Oehm E, Els T, Spreer J, Kassubek J, Hetzel A (2002). Transcranial color-coded sonography in basilar artery stenting. Ultrasound Med Biol.

